# Identification of a serum circulating lncRNA panel for the diagnosis and recurrence prediction of bladder cancer

**DOI:** 10.18632/oncotarget.12880

**Published:** 2016-10-25

**Authors:** Weili Duan, Lutao Du, Xiumei Jiang, Rui Wang, Suzhen Yan, Yujiao Xie, Keqiang Yan, Qingliang Wang, Lili Wang, Xin Zhang, Hongwei Pan, Yongmei Yang, Chuanxin Wang

**Affiliations:** ^1^ Department of Clinical Laboratory, Qilu Hospital of Shandong University, Jinan, 250012, Shandong Province, China; ^2^ Department of Urology, Qilu Hospital of Shandong University, Jinan, 250012, Shandong Province, China; ^3^ Department of Medical Affairs Management, Qilu Hospital of Shandong University, Jinan, 250012, Shandong Province, China

**Keywords:** lncRNA, bladder cancer, diagnosis, recurrence, serum

## Abstract

Accumulating evidence indicates that long non-coding RNAs (lncRNAs) play important roles in tumorigenesis and progression. We aimed to identify a panel of lncRNAs for the diagnosis and recurrence prediction in bladder cancer (BC). The expression of 13 candidate lncRNAs was investigated in 80 BC and matched adjacent normal tissues via quantitative real-time PCR. The differentially expressed lncRNAs were then analyzed in 240 serum samples (training set) and three lncRNAs (MEG3, SNHG16 and MALAT1) showed differential expression. A logistic regression model was constructed using the training set and validated in an independent cohort of 200 serum samples (validation set). The AUC of the three-lncRNA panel was 0.865 for the training and 0.828 for the validation set. The diagnostic performance of the lncRNA panel for Ta, T1, and T2–T4 were 0.778, 0.805, and 0.880, which were significantly higher than those of urine cytology (0.548, 0.604, and 0.682, respectively). Moreover, we determined that low expression of MEG3 was associated with poor recurrence-free survival by Kaplan-Meier analysis (*p* = 0.028), univariate Cox analysis (*p* = 0.033) and multivariate Cox analysis (*p* = 0.046). In conclusion, our results identified a three-lncRNA panel for BC diagnosis and a recurrence-independent prognostic factor, MEG3.

## INTRODUCTION

Bladder cancer (BC) is one of the most common urogenital malignancies worldwide, and is characterized by a high recurrence rate [1, 2]. Early screening and monitoring are essential for early and improved treatment of BC. Cystoscopy and cytology are currently the standard modalities used to diagnose and monitor urothelial carcinoma. However, cystoscopy is invasive, costly and often associated with discomfort; voided urine cytology lacks the diagnostic sensitivity necessary to rule out cancer. Thus, more reliable non-invasive makers of bladder cancer are urgently needed. Many urine-based biomarkers, such as bladder tumor antigen (BTA), nuclear matrix protein 22 (NMP22) and cytokeratin, have been developed during the past decades, but all of them have lower specificity than cytology, and none of them is recommended for large-scale cancer screening [3].

Long non-coding RNAs (lncRNAs) are a class of transcripts with more than 200 nucleotides and limited or no protein coding capacity. Accumulating evidence is supporting the involvement of lncRNAs in tumorigenesis and progression in various types of cancers including BC by modulating oncogenic and tumor-suppressing pathways. The aberrantly expressed lncRNAs have been reported as the potential biomarkers for the diagnosis, prognosis and target therapy of cancers [4]. Although these lncRNAs have revealed the great promising as tissue-based markers for cancers, which will constitute an invasive procedure with possible complications, costly and prone to sampling errors.

Circulating RNAs in blood have been described as cancer biomarkers for noninvasive diagnosis [5]. The detection and identification of lncRNAs in serum or plasma may provide a new realm for early diagnosis and treatment of tumors. Recently, a few circulating lncRNAs have been explored as promising biomarkers for the diagnosis of tumors. For instance, plasma H19 may serve as a potential biomarker to diagnose gastric cancer and monitor tumor dynamics for tumor resection [6]. In patients with esophageal squamous cell carcinoma (ESCC), the combination of plasma POU3F3 and SCCA can provide high diagnostic performance for ESCC, and also indicated that plasma and serum were both acceptable for lncRNAs analysis as blood-based biomarkers [7]. The serum three-lncRNA signature including CUDR, LSINCT-5 and PTENP1 may be a more accurate biomarker than CEA and CA19-9 for the diagnosis of gastric cancer and facilitate the detection of gastric cancer at the early stage [8]. However, there are no systematic reports on the possible application of circulating lncRNA quantification in patients with BC.

In the present study, 13 lncRNAs (MEG3, SNHG16, MALAT1, PCAT-1, GHET, H19, UBC1, SPRY4-IT1, TUG1, UCA1, lincRNA-PRss16, BC039493 and GAS5) were selected as the candidate lncRNAs [9–20] because their dys-regulation has been previously reported in BC. Then, we performed an initial screening using quantitative real-time PCR (qRT-PCR) to evaluate the expression of lncRNAs in tissues followed by a two-step investigation for comprehensive evaluation of lncRNA concentrations in serum. Meanwhile, a three-lncRNA panel with high diagnostic performance was explored for the detection of BC. Finally, we also examined the association between three lncRNAs and BC recurrence.

## RESULTS

### Selection and investigation of BC-related lncRNAs in tissues

On the basis of previous studies, 13 lncRNAs with differential expression in BC were selected as the candidate lncRNAs in our study. The relative expression levels of 13 lncRNAs were measured by qRT-PCR in 80 BC samples and matched adjacent normal tissues. The nonparametric Mann-Whitney *U* tests for the identification revealed that 11 of 13 lncRNAs were significantly dys-regulated between bladder samples and matched adjacent normal tissues. However, GAS5 and BC039493 showed no statistically differential expression between two groups and were excluded in the subsequent study (Supplementary Table S2).

### Evaluation of eleven lncRNAs expression in serum

**Figure 1:**
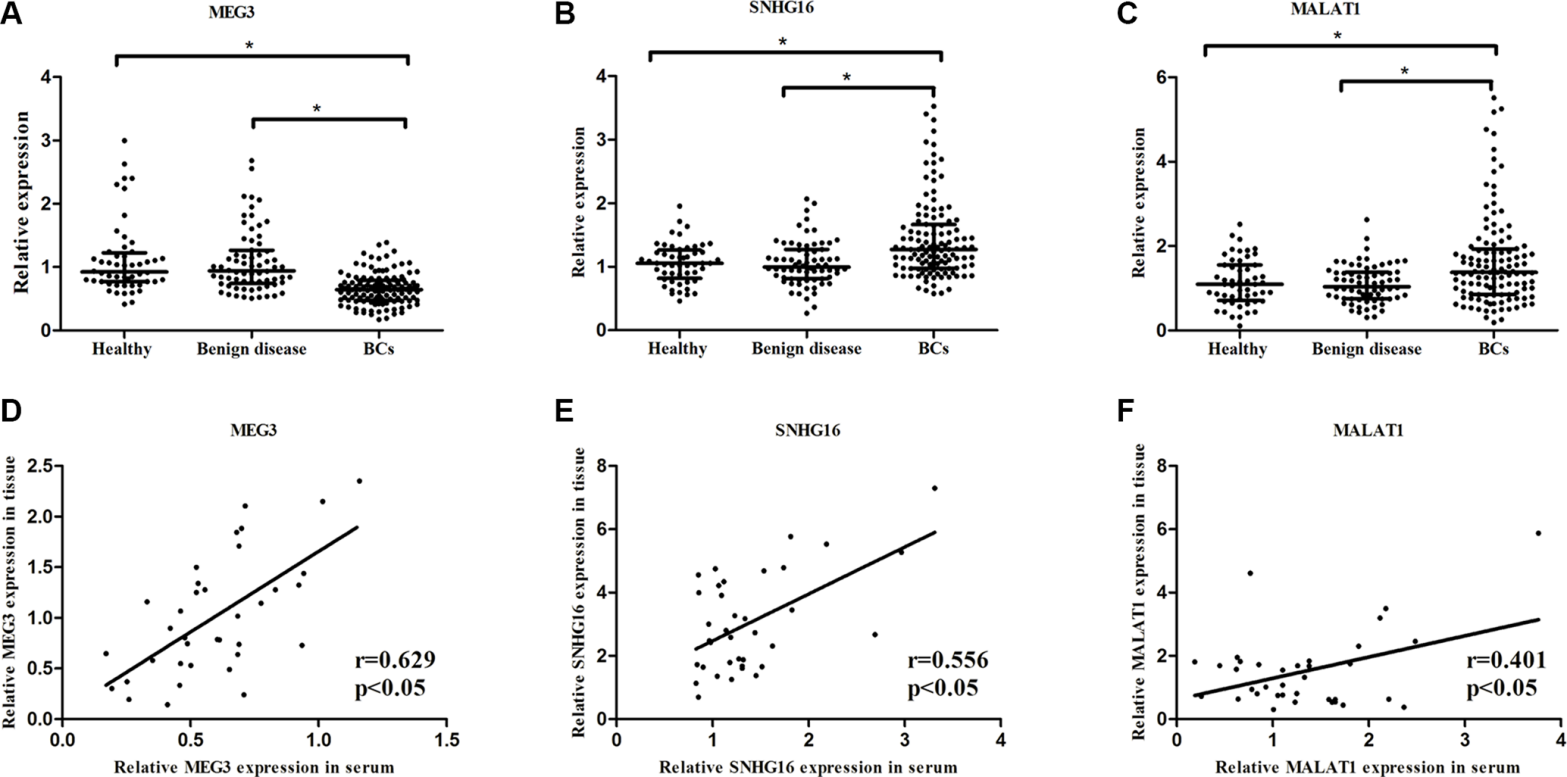
Expression levels of serum MEG3, SNHG16 and MALAT1 and their expression in paired serum and tissue Expression levels of serum MEG3 (**A**), SNHG16 (**B**) and MALAT1 (**C**) in Healthy vs. Benign disease (*p >* 0.05), Healthy vs. BCs (*p <* 0.01) and Benign disease vs. BCs comparisons (*p <* 0.01). The scatter plot showed the relative expression of MEG3 (**D**), SNHG16 (**E**) and MALAT1 (**F**) in BC tissues and serum. Date were presented as 2^−ΔΔCt^.

**Figure 2:**
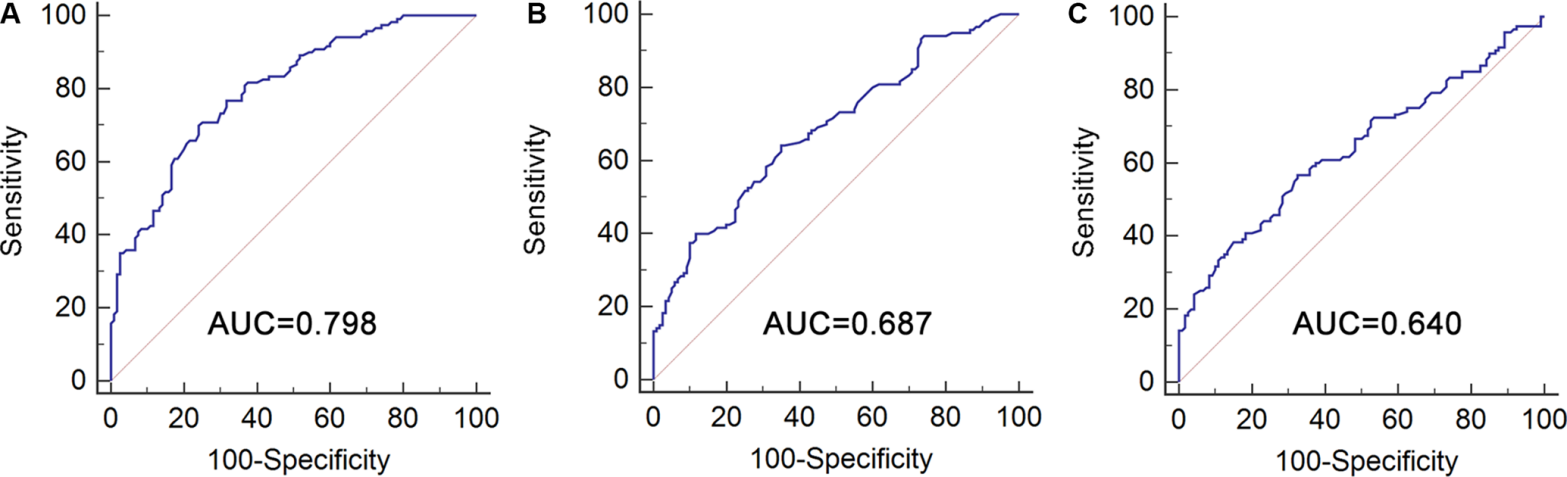
Diagnostic performance of selected lncRNAs for BC patients versus controls Receiver operating characteristics (ROC) curve analysis for detection of BC using MEG3 (**A**), SNHG16 (**B**) and MALAT1 (**C**) in BC patients and controls in training set.

The selected eleven candidate lncRNAs were first evaluated by qRT-PCR technology in serum samples from 52 healthy subjects, 68 benign disease and 120 BC patients in the training set. Consequently, only three lncRNAs (MEG3, SNHG16 and MALAT1) showed a statistically differential expression in healthy vs. BCs and benign disease vs. BCs comparisons (Table 1). MEG3 was significantly down-regulated, SNHG16 and MALAT1 were significantly up-regulated in healthy vs. BCs and benign disease vs. BCs comparisons (Figure 1A–1C). The corresponding AUCs of the three lncRNA to distinguish BC patients from controls were 0.798 (95% *CI* = 0.741–0.846, sensitivity = 70.0% and specificity = 75.8%), 0.687 (95% *CI* = 0.624−0.745, sensitivity = 64.2% and specificity = 65.0%), and 0.640 (95% *CI* = 0.576−0.701, sensitivity = 56.7% and specificity = 67.5%), respectively (Figure 2). In order to verify the accuracy and specificity of these three lncRNAs (MEG3, SNHG16 and MALAT1) as the BC signature, we assessed their expression levels using another independent sample set containing 48 healthy subjects, 52 benign disease and 100 BC patients (Supplementary Figure S1 and Supplementary Figure S2). The changing trend of the expression of 3 lncRNAs was generally concordant between training set and validation set, and revealed no significant difference (Table 1).

**Table 1:** The selected serum lncRNA concentrations in healthy vs. benign disease, healthy vs. BCs, and benign disease vs. BCs comparisons in training set and validation set [median (interquartile range)]

	Categories	MEG3	*P*-value	SNHG16	*P*-value	MALAT1	*P*-value
**Training set**							
	Healthy vs. Benign disease	0.93 (0.77–1.23) vs. 0.94 (0.74–1.27)	> 0.05	1.06 (0.82–1.26) vs. 1.00 (0.81–1.27)	> 0.05	1.10 (0.71–1.55) vs. 1.04 (0.75–1.38)	> 0.05
	Healthy vs. BCs	0.93 (0.77–1.23) vs. 0.64 (0.48–0.80)	< 0.01	1.06 (0.82–1.26) vs. 1.27 (0.98–1.67)	< 0.01	1.10 (0.71–1.55) vs. 1.38 (0.86–1.93)	< 0.01
	Benign disease vs. BCs	0.94 (0.74–1.27) vs. 0.64 (0.48–0.80)	< 0.01	1.00 (0.81–1.27) vs. 1.27 (0.98–1.67)	< 0.01	1.04 (0.75–1.38) vs. 1.38 (0.86–1.93)	< 0.01
**Validation set**							
	Healthy vs. Benign disease	1.05 (0.75–2.07) vs. 0.97 (0.67–1.21)	> 0.05	0.97 (0.63–1.31) vs. 1.04 (0.82–1.47)	> 0.05	1.15 (0.57–1.69) vs. 1.19 (0.91–1.50)	> 0.05
	Healthy vs. BCs	1.05 (0.75–2.07) vs. 0.54 (0.37–0.81)	< 0.01	0.97 (0.63–1.31) vs. 1.28 (1.03–1.55)	< 0.01	1.15 (0.57–1.69) vs. 1.39 (1.06–1.81)	< 0.01
	Benign disease vs. BCs	0.97 (0.67–1.21) vs. 0.54 (0.37–0.81)	< 0.01	1.04 (0.82–1.47) vs. 1.28 (1.03–1.55)	< 0.01	1.19 (0.91–1.50) vs. 1.39 (1.06–1.81)	< 0.01

### Confirmation of serum lncRNAs as the biomarkers

In order to explore the potential role of circulating cell-free lncRNAs as the biomarkers for the diagnosis of BC, we first analyzed the correlation of MEG3, SNHG16 and MALAT1 expression levels between 36 serum samples and corresponding tumor tissue samples. A significant correlation was observed for MEG3 (r = 0.629, *p <* 0.05), SNHG16 (r = 0.556, *p <* 0.05) and MALAT1 (r = 0.401, *p <* 0.05), respectively (Figure 1D–1F).

We next investigated the stability of serum MEG3, SNHG16 and MALAT1. The serum samples from 5 patients with BC were exposed to harsh conditions including incubation at room temperature for 4, 8, and 24 h or incubation at −80°C for 1, 2, and 3 months, or 2, 4, and 8 repetitive freeze-thaw cycles. Results indicated that these treatments had no any effects on serum levels of MEG3, SNHG16 and MALAT1, which provides a base for cancer diagnosis as the useful and stable biomarkers (Supplementary Figure S3).

### Establishment of the predictive lncRNA panel

**Figure 3:**
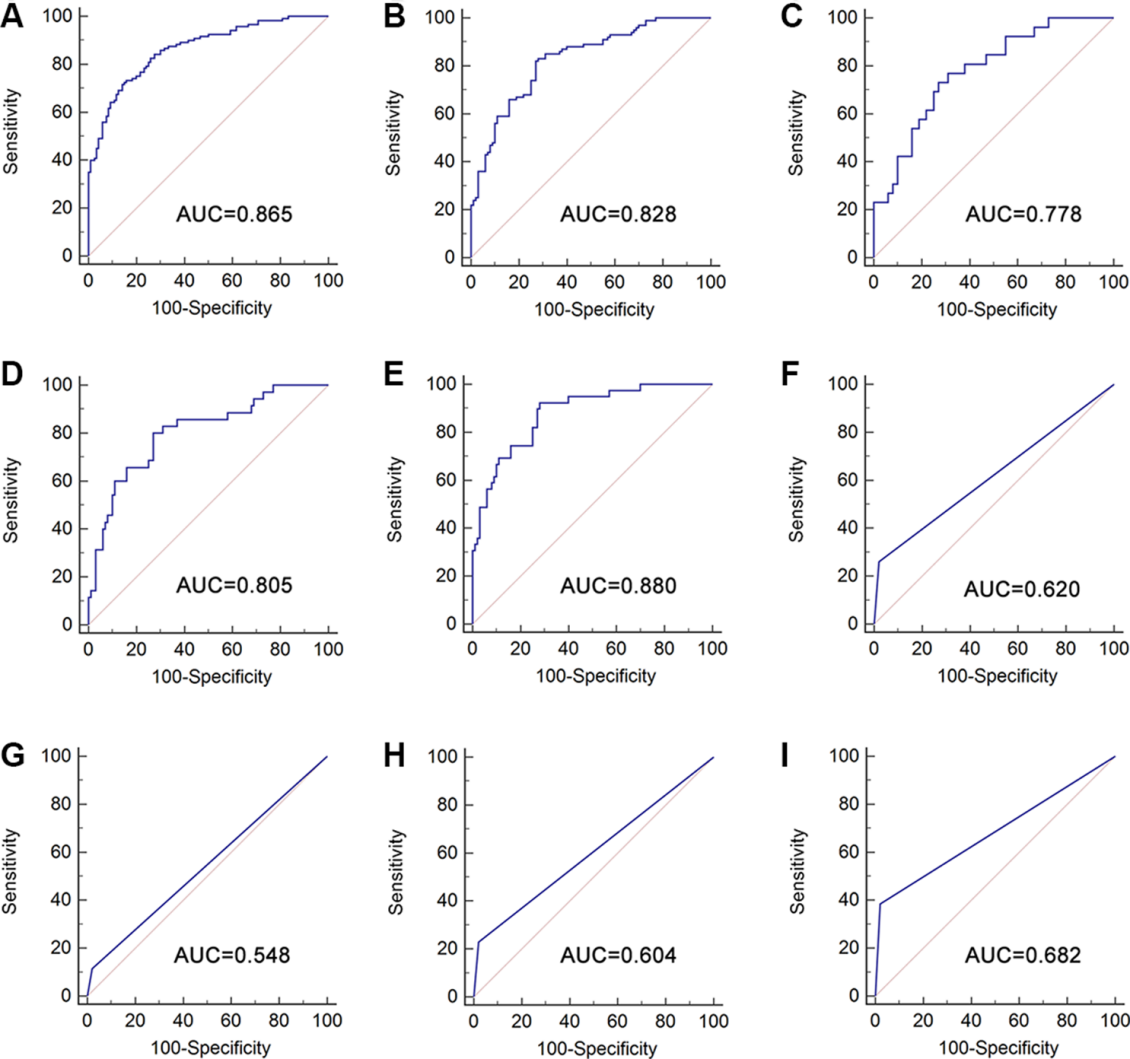
Diagnostic performance of three-lncRNA panel and urine cytology for the detection of BC Receiver operating characteristics curve (ROC) analysis using three-lncRNA panel for the detection of BC in training set (**A**) and in validation set (**B**); ROC curves showing the diagnostic performance of the three-lncRNA panel for Ta (**C**), T1 (**D**) and T2–T4 (**E**) in validation set; ROC curve analysis for the detection of BC using urine cytology for the detection of BC with all stages (**F**), Ta (**G**), T1 (**H**), and T2–T4 (**I**) in validation set.

Through the training date set, a stepwise logistic regression model was established to estimate the risk of being diagnosed as BC. The predicted probability of BC from the logit model based on the three-lncRNA panel, logit (*p* = BC) = 0.0904 + 0.929 * MEG3 – 0.5094 * SNHG16 – 0.1986 * MALAT1 was used to construct the ROC curve. To evaluate the performance of the established lncRNA-panel for the diagnosis of BC, AUC analysis was carried out. The AUC for the lncRNA panel was 0.865 (95% *CI* = 0.815–0.905, sensitivity = 71.7% and specificity = 85.8%) (Figure 3A).

### Validation of the lncRNA panel

The parameters estimated from the training date set were entered into another cohort of 200 participants containing 100 patients with BC and 100 controls to predict the probability of being diagnosed as BC. Similarly, AUC analysis was performed to determine the capacity of the lncRNA panel to distinguish BC patients from the controls. The AUC of the three-lncRNA panel was 0.828 (95% *CI* = 0.768−0.877, sensitivity = 82.0% and specificity = 73.0%) (Figure 3B). The AUCs of the panel for BC patients diagnosed as Ta, T1 and T2–T4 were 0.778 (95% *CI* = 0.696–0.848, sensitivity = 73.1% and specificity = 73.0%), 0.805 (95% *CI* = 0.728−0.868, sensitivity = 80.0% and specificity = 73.0%) and 0.880 (95% *CI* = 0.814–0.929, sensitivity = 92.3% and specificity = 72.0%), respectively (Figure 3C–3E).

The comparison of the diagnostic performance between the three-lncRNA panel and urine cytology for distinguishing patients with BC from controls was performed in the validation set. The AUC of urine cytology for the detection of BC at all stages was 0.620 (95% *CI* = 0.549−0.688, sensitivity = 26.0% and specificity = 98.0%) (Figure 3F). The AUCs of urine cytology for Ta, T1 and T2–T4 were 0.548 (95% *CI* = 0.457−0.636, sensitivity = 11.5% and specificity = 98.0%), 0.604 (95% *CI* = 0.517−0.687, sensitivity = 22.9% and specificity = 98.0%) and 0.682 (95% *CI* = 0.598−0.759, sensitivity = 38.5% and specificity = 98.0%), respectively (Figure 3G–3I), which were significantly lower than those from the three-lncRNA panel.

### Correlation between three lncRNAs and clinicopathological characteristics

The relationship between three lncRNAs and clinicopathological characteristics of the patients with BC in the validation set was summarized in Supplementary Table S3. Lower level of MEG3 was significantly correlated with advanced tumor stage (*p* = 0.03). However, there was no significant association between three lncRNAs with age, sex, tumor grade and positive lymph node metastasis (all at *p* > 0.05).

### Association of recurrence free survival with three serum lncRNAs

**Figure 4:**
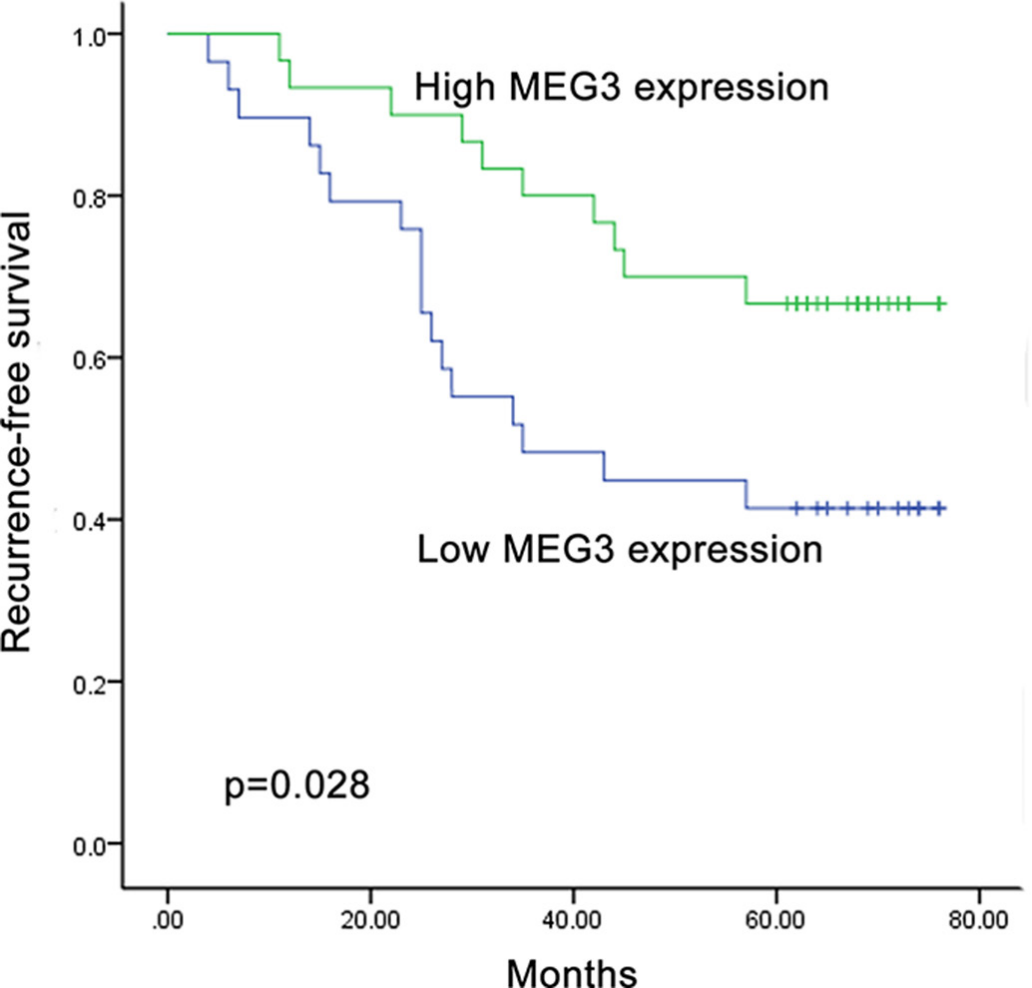
Recurrence prediction of MEG3 expression Kaplan-Meier curve showed that low level of serum MEG3 was associated with a worse recurrence-free survival in NMIBC patients in validation set.

In the validation set (*n* = 100), five patients including two NMIBC and three MIBC were excluded during the follow-up period due to the incomplete records. Kaplan-Meier survival analysis was carried in the NMIBC group (*n* = 59). We observed that patients with low MEG3 level had shorter recurrence-free survival (RFS) (*p* = 0.028) (Figure 4). However, SNHG16 and MALAT1 expression level had no correlation with RFS of BC. Univariate analysis using the Cox proportional hazard regression model revealed a statistically significant correlation between RFS of NMIBC and MEG3 level (*p* = 0.033), and tumor stage (*p* = 0.030). Multivariate analysis including MEG3 and tumor stage showed that MEG3 (*p* = 0.046) and tumor stage (*p* = 0.041) were independent prognostic factors for the RFS of NMIBC (Table 2). In the MIBC group (*n* = 36), however, none of three dys-regulated lncRNAs influenced predicted recurrence of the patients (all at *p* > 0.05, Supplementary Table S4).

**Table 2:** Univariate and multivariate Cox proportional hazards regression model analysis of RFS in patients with NMIBC in validation set

Paraments	Categories	Univariate analysis		Multivariate analysis	
		HR (95%CI)	*P*-Value	HR (95%CI)	*P*-Value
Age	< 67 vs. ≥ 67	0.966 (0.454–2.057)	0.929		
Sex	Male vs. female	0.944 (0.399–2.233)	0.895		
Tumor stage	Ta vs. T1	2.502 (1.091–5.736)	0.030	2.378 (1.036–5.461)	0.041
Tumor grade	Low vs. high	1.023 (0.468–2.236)	0.954		
Lymph node metastasis	Negative vs. positive	1.044 (0.247–4.414)	0.954		
MEG3 expression	Low vs. high	0.427 (0.195–0.934)	0.033	0.450 (0.205–0.987)	0.046
SNHG16 expression	Low vs. high	1.632 (0.762–3.497)	0.208		
MALAT1 expression	Low vs. high	0.613 (0.284–1.322)	0.212		

Abbreviations: RFS, Recurrence-free survival; HR, Hazard ratio; CI, Confidence interval.

## DISCUSSION

In the current study, we systematically examined the expression levels of lncRNAs in pooled tumor and matched adjacent normal tissues combined with individual qRT-PCR validation in serum samples. Then, a three-lncRNA panel containing MEG3, SNHG16 and MALAT1 was selected as a novel diagnostic biomarker for BC. Furthermore, ROC analysis was performed to assess the diagnostic performance of the three-lncRNA panel, suggesting that circulating three-lncRNA panel can differentiate BC patients from controls and has superior diagnostic performance than urine cytology. In addition, among these three lncRNAs, circulating MEG3 was proved to be an independent prognostic factor for RFS.

Functional studies of lncRNAs in tumor tissues may be helpful for evaluating serum lncRNAs as the indicators of various types of cancers. These 3 lncRNAs have been previously reported as the aberrantly expressed lncRNAs in BC and other cancer tissue samples. Greife et al. [21] have also demonstrated that MEG3 is down-regulated in BC tissues, and is related to the acquisition of novel DNA methylation patterns, especially at the differently methylated region of MEG3. Moreover, Ying et al. [9] have determined that the down-regulation of MEG3 can inhibit BC cell apoptosis and increase cell proliferation by activating autophagy. The overexpression of SNHG16 (ncRAN) is positively correlated with aggressive bladder cancer and the silencing of SNHG16 can improve chemotherapy sensitivity in BC cell lines [10]. Contrast to the expression tendency in BC tissues, SNHG16 is down-regulated in colorectal cancer tissue [22]. As an oncogene, MALAT1 is strongly up-regulated in BC tissues when compared with adjacent normal tissues and contributes to BC cell migration by inducing epithetlial-to-mesenchymal transition [11]. In addition, the expression level of MALAT1 is associated with the overall survival (OS) of BC [23]. The overexpression of MALAT1 has also been described in gastric cancer [24], prostate cancer [25] and colorectal cancer [26]. As mentioned above, the biomarkers we have discovered should be involved in the development of BC and many other cancers, suggesting a primary role of these three lncRNAs in bladder carcinogenesis.

To date, among these 3 lncRNAs identified in our study, none have been reported their dys-regulation in the plasma or serum from BC patients. What's more, we have confirmed that serum lncRNAs are stable even treated with longer incubation time at room temperature or at −80°C and multiple freeze-thaw cycles. One possible explanation for the remarkable stability of circulating cell-free lncRNAs is due to the protection by extracellular vesicles including exosomes, microvesicles and apoptotic bodies [27–29], while another possible explanation is that lncRNAs can fold into complex secondary and high-order structures or combination with protein that similar to miRNAs [4, 30, 31]. In addition, a moderate significant correlation of MEG3, SNHG16 and MALAT1 between bladder cancer tissues and paired serum samples was also observed, which provides the strong evidence that BC-related lncRNAs could be released into the circulation and enriched in serum. Therefore, circulating lncRNAs in serum may reflect, at least partially, its status in BC tissues.

Previous studies on the screening of potential lncRNA biomarkers are roughly focused on the single lncRNA. However, due to the complex pathogenesis during initiation and progression of severe malignancy, a signal lncRNA may be an unreliable biomarker to detect tumors timely. In this regard, the development of a lncRNA panel in serum may improve the diagnostic performance for tumors. Here, after a stepwise selection, we have identified a circulating lncRNA panel containing three lncRNAs (MEG3, SNHG16 and MALAT1) with the AUCs of 0.789, 0.679, and 0.635, respectively. However, the combination of three lncRNAs has an AUC of 0.828, which is significantly improved when compared with MEG3, SNHG16, or MALAT1 alone. In addition, the three-lncRNA panel also had higher sensitivity to detect Ta and T1 tumors and revealed the detection sensitivity of 73.1% and 80.0%, respectively, while urine cytology with the detection sensitivity of only 11.5% and 22.9% in the same cohort. These results demonstrate that the three-lncRNA panel may be a potential minimally-invasive biomarker for the diagnosis of BC, especially at the early stage.

In the present study, we have also focused on the correlation between three lncRNAs and the recurrence of BC. The Kaplan-Meier analysis has identified the decreased expression of serum MEG3 with significant association with poorer prognosis in terms of NMIBC RFS. Furthermore, we have confirmed that serum MEG3 was an independent risk factor for RFS in NMIBC through univariate and multivariate analysis. However, no dys-regulated lncRNAs can result in the impact on RFS of patients with MIBC. Although, MEG3 is identified for the first time as an excellent indicator of BC prognosis, other studies have described the similar findings in several other cancers. Yin et al. has shown that MEG3 can serve as an independent predictor for OS of colorectal cancer [32]. Moreover, MEG3 also can be identified as an independent prognostic factor for RFS and OS of hepatocellular carcinoma patients [33], which notably suggests a major role of MEG3 in the prediction of prognosis. These results demonstrate that circulating MEG3 status in tumors may be a useful tool for estimating prognosis of patients with BC and for selecting patients who are likely to benefit from adjuvant therapy to reduce the risk of recurrence.

In summary, serum lncRNAs were capable of identifying patients suffering from BC. In addition, MEG3 was proved to be an independent predictor for the recurrence of NMIBC. Although the preliminary nature of the study is warranted in multicenter validation, our findings should be set in various stages for exploring the clinical value of serum lncRNAs as the potential biomarkers for the diagnosis of BC and the predictors for the prognosis of BC.

## MATERIALS AND METHODS

### Study design

In this study, we selected 13 lncRNAs from previously published studies as the candidate lncRNAs. The expression levels of these 13 candidate lncRNAs were initially measured by qRT-PCR in 80 BC samples and matched adjacent normal tissues; among these lncRNAs, 11 lncRNAs were differentially expressed and further investigated in serum samples including training set and validation set. In the training set, we first investigated the expression of 11 lncRNAs through qRT-PCR in an independent cohort from 52 healthy subjects, 68 benign disease (32BPH, 16 Urolithiasis, 20 Cystitis) and 120 BC patients. Among them, three lncRNAs were significantly dys-regulated in healthy vs. BCs and benign disease vs. BCs comparisons, but no statistical different expression was found between healthy and benign disease. The other 8 lncRNAs showed no significant change in healthy vs. benign disease, healthy vs. BCs and benign disease vs. BCs comparisons. Then, we used the training set to construct the diagnostic lncRNA panel on the basis of logistic regression model for the differentiation between BC patients and controls (healthy and benign disease). Afterwards, the stability of three lncRNAs in serum samples from five patients and the comparison of MEG3, SNHG16 and MALAT1 expression levels between serum and corresponding cancer tissues from 36 patients were analyzed, respectively. In the validation set, the parameters of the logistic model from the training set were entered into another cohort of 200 participants including 48 healthy subjects, 52 benign disease (21BPH, 15 Urolithiasis, 16 Cystitis) and 100 patients with BC, to verify the diagnostic performance of the selected lncRNA panel. Additionally, urine cytology was applied to urine samples from the same cohort.

In the validation set, the patients who were free of disease were assessed every 3 months during the first 2 years and then every 6 months. The date of latest record retrieved was September 30, 2015. The Kaplan-Meier survival analysis was applied for 59 NMIBC and 36 MIBC, while 5 patients including 2 NMIBC and 3 MIBC were excluded because of incomplete follow-up date. The median follow-up time was 57 months (range, 4–76 months).

### Sample collection

During this study, tissue, serum and urine samples with eligibility criteria were collected from Qilu Hospital of Shandong University between 2007 and 2015. Eighty tumor tissues and matched adjacent normal tissue samples were obtained from patients who had undergone transurethral bladder resection (TUR or radical cystectomy. No patients were subjected to preoperative radiotherapy, chemotherapy or other cancer treatments. All these tissue samples were immediately frozen in liquid nitrogen and then stored at −80°C until total RNA extraction.

A total of 220 serum samples enrolled in the present study were harvested from patients with BC. Serum samples were also harvested from 220 volunteer donors (53BPH, 31 Urolithiasis, 36 Cystitis and 100 healthy) as the control group. Clinical and demographic features of BC patients and controls were shown in Supplementary Table S1. All these healthy subjects, benign disease and BC patients showed no evidence of disease in other organs. The serum was seperated from venous blood within 2 h using a two-step centrifugation protocol (4000 rpm for 10 min at 4°C, and 12000 rpm for 15 min at 4°C) to thoroughly remove cell debris. Each supernatant was transferred into RNase and DNase free tubes and stored at −80°C until future use.

Urine samples were collected before cystoscopic examination and any other treatments, and centrifuged at 1300 g for 10 min. The sediments were used for cytological analysis and the diagnosis was confirmed by two cytopathologists.

BC patients were diagnosed by histobiopsy or histopathology, while tumors were staged and graded according to the tumor-node metastasis (TNM) staging system and the WHO 2004 grading scheme, respectively. The study was approved by QiLu Hospital, Shandong University. Written informed consent was obtained for the use of all patient samples. All procedures were performed in accordance with relevant regulations and guidelines.

### RNA extraction and qRT-PCR

RNA extraction from tissue samples was performed using Trizol (Invitrogen, Carlsbad, CA) Reagent whereas total RNA in serum was isolated by using Trizol LS (Invitrogen, Carlsbad, CA) following the manufacturer's protocol. The concentration of RNA was measured by NanoDrop spectrophotometer. The PrimeScript^TM^RT reagent kit (Takara, Dalian, Liaoning) was used for reverse transcription of the lncRNA. After mixing with 1 μg of template RNA, 4 μL of 5× PrimeScript Buffer, 1 μL of PrimeScript RT Enzyme Mix I, 1 μL of Oligo dT Primer, and RNase-free dH_2_O in a final volume of 20 μL, the mixture was centrifuged briefly and incubated at 37°C for 30 min, followed by 85°C for 5 s and 4°C for 60 min. The quantitative polymerase chain reaction was carried out in a volume of 25 mL containing 12.5 μL of SYBR Premix Ex Taq, 0.5 μL of ROX Reference Dye α, 1 μL of forward primer (10 μM), 1 μL of reverse primer (10 μM), 2 μL of cDNA product and 8 μL of RNase-free dH_2_O in the CFX-96 real-time PCR System using the SYBR^®^ Premix Ex Taq^TM^ (Takara, Dalian, Liaoning). The reaction mixture was incubated at 95°C for 30 s, followed by 42 cycles at 95°C for 5 s, 60°C for 34 s. All reactions were carried out in triplicate. The melting curve was used to determine the specificity of the qRT-PCR product. The relative expression of lncRNAs from serum and tissue samples was calculated using the 2^−^^ΔΔCT^ method and normalized using the GAPDH as the reference.

### Statistical analysis

Nonparametric Mann-Whitney *U* tests were employed to compare the expression levels of tissue and serum lncRNAs between BC patients and controls. Receiver operating characteristic (ROC) curve was established for discriminating BC patients and controls using MedCala 9.3.9.0 (MedCala, Mariakerke, Belgium). Area under the ROC curve (AUC) was used to evaluate the diagnostic performance of the selected lncRNA panel. Survival curves of NMIBC and MIBC were established by Kaplan-Meier method, respectively, and the difference was assessed using log-rank statistics. Cox regression multivariate analysis was performed to estimate the independent prognostic factors for recurrence prediction. Statistical calculations were performed using SPSS version 19.0 software (SPSS, Chicago, IL). A *p*-value less than 0.05 was considered as statistically significant difference.

## SUPPLEMENTARY MATERIALS


