# Supplementary material for: Identification of a serum circulating lncRNA panel for the diagnosis and recurrence prediction of bladder cancer
Source: Oncotarget. 2016 Oct 25;7(48):78850–8. doi: 10.18632/oncotarget.12880 (PMC5346682; doi:10.18632/oncotarget.12880)
Supplement: Supplementary file 1 [file oncotarget-07-78850-s001.pdf]

Identification of a serum circulating lncRNA panel for the diagnosis and recurrence prediction of bladder cancer

Supplementary Materials

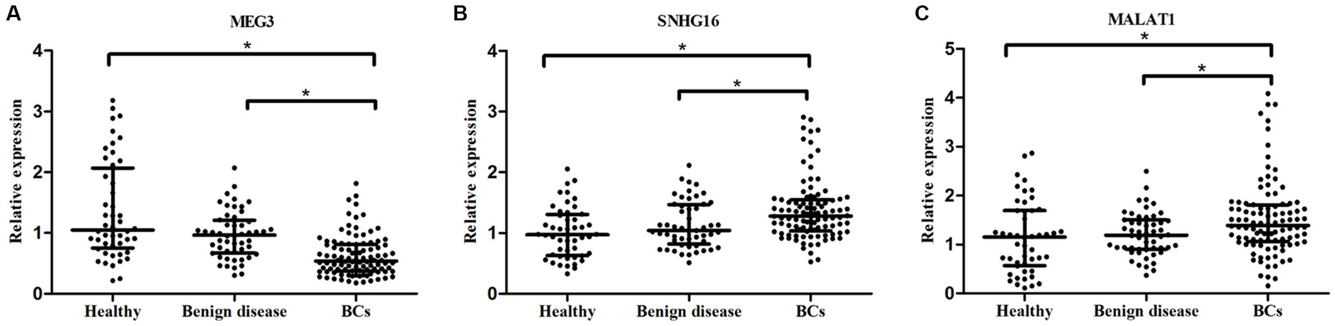

Supplementary Figure S1: Validation of MEG3, SNHG16 and MALAT1 as biomakers for detecting BC. Expression levels of three selected lncRNAs (MEG3, SNHG16 and MALAT1) in serum samples in Healthy vs. Benign disease ($p > 0.05$), Healthy vs. BCs ($p < 0.01$) and Benign disease vs. BCs comparisons ($p < 0.01$) in validation set.

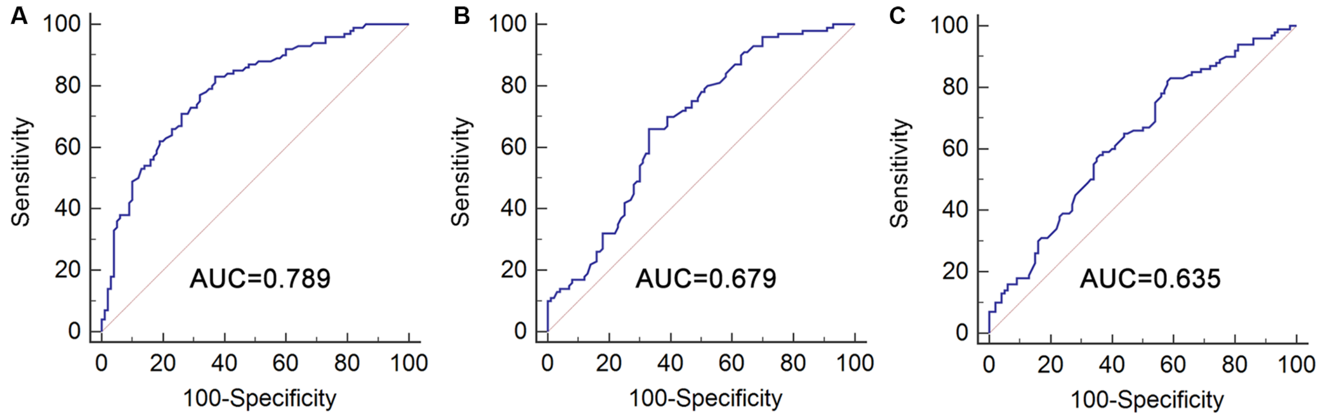

Supplementary Figure S2: Validation of diagnostic performance of selected lncRNAs for BC patients versus controls. Receiver operating curves indicated the ability of the serum levels of three individual lncRNAs including MEG3 (**A**), SNHG16 (**B**) and MALAT1 (**C**) to differentiate BC patients from contols in validation set.

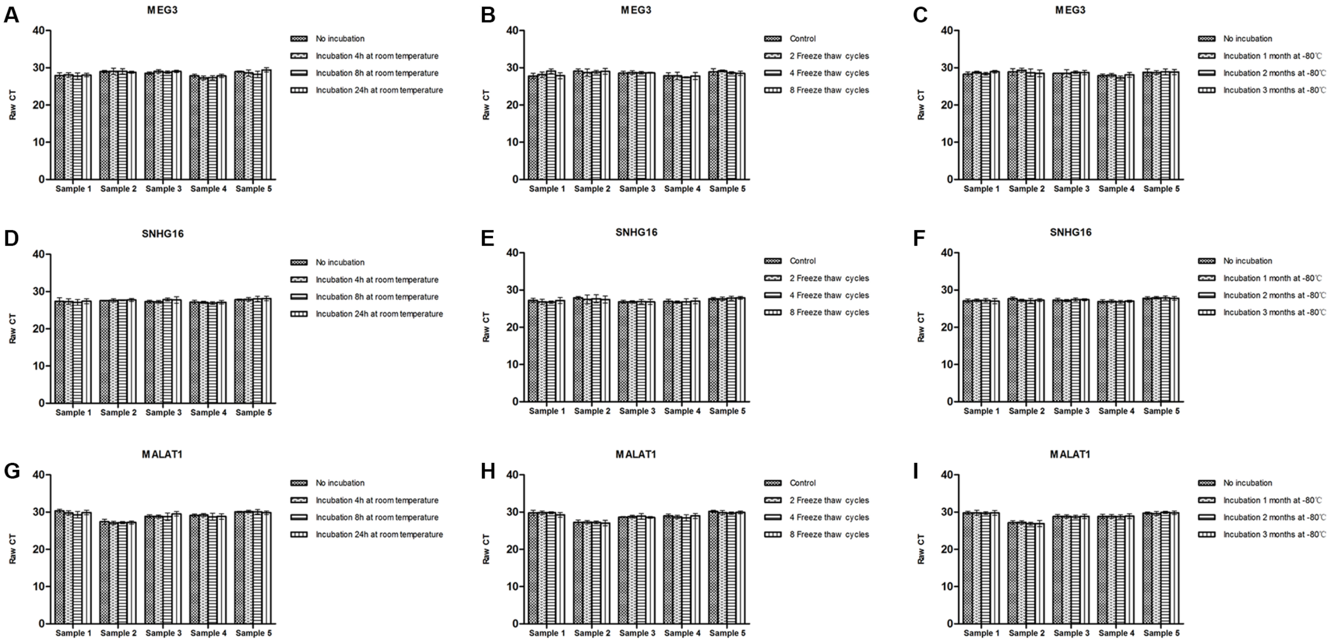

Supplementary Figure S3: Stability of serum MEG3, SNHG16 and MALAT1. MEG3, SNHG16, and MALAT1 serum levels remained stable when treated with prolonged exposure to room temperature, or multiple freeze-thaw cycles, or prolonged −80°C incubation time.

Supplementary Table S1: Characteristics of study participants in training set and validation set [median (interquartile range)]

Variable	Training set	Validation set	*P*-Value
	n = 240	*n* = 200	
**Contronl (number)**	120	100	
**Age (years)**			0.33
≤ 60	46 (38.33%)	32 (32%)	
> 60	74 (61.67%)	68 (68%)	
**Sex**			0.71
Male	96 (80%)	82 (82%)	
Female	24 (20%)	18 (18%)	
**Healthy (number)**	52 (43.33%)	48 (48%)	
**Benign disease**			0.73
BPH	32 (26.67%)	21 (21%)	
Urolithiasis	16 (13.33%)	15 (15%)	
Cystitis	20 (16.67%)	16 (16%)	
**BC (number)**	120	100	
**Age (years)**			0.34
≤ 60	42 (35%)	29 (29%)	
> 60	78 (65%)	71 (71%)	
**Sex**			0.30
Male	97 (80.83%)	75 (75%)	
Female	23 (19.17%)	25 (25%)	
**Tumor stage**			0.63
Ta–T1	77 (64.17%)	61 (61%)	
T2–T4	43 (35.83%)	39 (39%)	
**Tumor grade**			0.59
Low grade	68 (56.67%)	53 (53%)	
High grade	52 (43.33%)	47 (47%)	
**Lymph node matastasis**			0.55
Negative	101 (84.17%)	87 (87%)	
Positive	19 (15.83%)	13 (13%)	

Abbreviations: BPH, benign prostatic hyperplasia; BC, bladder cancer.

Supplementary Table S2: The selected lncRNA concentrations in BC tissues as compared with matched adjacent normal tissues [median (interquartile range)]

lncRNA	Controls	BCs	*P*	lncRNA	Controls	BCs	*P*
MEG3	1.09 (0.53–2.08)	0.78 (0.50–1.27)	< 0.01	SPRY-IT1	0.97 (0.59–1.64)	3.10 (1.26–5.78)	< 0.01
SNHG16	1.26 (0.57–1.80)	3.10 (2.34–3.98)	< 0.01	TUG1	1.02 (0.56–2.15)	5.28 (3.64–7.23)	< 0.01
MALAT1	1.32 (0.54–2.17)	1.83 (0.81–3.21)	< 0.01	UCA1	1.10 (0.67–1.95)	2.37 (1.40–4.54)	< 0.01
PCAT-1	1.23 (0.44–2.08)	2.64 (1.47–5.52)	< 0.01	lincRNA-PRss16	0.99 (0.57–1.70)	1.32 (1.03–1.88)	< 0.01
GHET	1.25 (0.73–1.72)	1.55 (1.12–2.87)	< 0.01	GAS5	1.07 (0.65–1.60)	1.01 (0.52–1.76)	0.53
H19	1.13 (0.48–2.11)	2.32 (1.52–3.83)	< 0.01	BC039493	1.00 (0.71–1.41)	1.17 (0.76–1.59)	0.16
UBC1	1.07 (0.56–1.76)	3.92 (2.54–6.24)	< 0.01				

Supplementary Table S3: Correlation between serum lncRNA concentrations and clinicopathological characteristics of patients with BC in validation set [median (interquartile range)]

Parameters	Total cases	MEG3	*P*	SNHG16	*P*	MALAT1	*P*
**Age**			0.84		0.85		0.48
≤ 66	52	0.54 (0.37–0.79)		1.28 (0.97–1.53)		1.44 (1.09–1.86)	
> 66	48	0.54 (0.37–0.82)		1.28 (1.06–1.58)		1.35 (1.00–1.73)	
**Sex**			0.43		0.97		0.36
Male	75	0.57 (0.38–0.82)		1.31 (1.03–1.50)		1.36 (1.01–1.74)	
Female	25	0.51 (0.36–0.72)		1.25 (1.04–1.58)		1.49 (1.16–1.85)	
**Tumor stage**			0.03		0.50		0.72
Ta–T1	61	0.60 (0.41–0.83)		1.25 (1.05–1.49)		1.43 (1.09–1.76)	
T2–T4	39	0.51 (0.31–0.69)		1.33 (1.02–1.57)		1.36 (1.05–1.87)	
**Tumor grade**			0.73		0.06		0.71
Low grade	53	0.50 (0.37–0.82)		1.17 (0.98–1.49)		1.40 (1.00–1.80)	
High grade	47	0.57 (0.36–0.76)		1.37 (1.20–1.57)		1.38 (1.09–1.87)	
**Lymph node matastasis**			0.14		0.42		0.52
Negative	87	0.55 (0.40–0.83)		1.28 (1.02–1.54)		1.43 (1.09–1.78)	
Positive	13	0.48 (0.29–0.71)		1.33 (1.17–1.62)		1.10 (0.99–2.10)	

Supplementary Table S4: Univariate Cox proportional hazards regression model analysis of recurrence-free survival in MIBC patients in validation set

Parameters	Categories	Univariate analysis	
		HR (95% CI)	*p* Value
MEG3	Low vs. High	0.682 (0.305–1.525)	0.352
SNHG16	Low vs. High	1.526 (0.683–3.411)	0.303
MALAT1	Low vs. High	1.833 (0.794–4.228)	0.156

Abbreviations: HR, hazard ratio; CI, confidence interval.
